# Metabolic Syndrome among Patients with Polycystic Ovarian Syndrome Presenting to a Tertiary Care Hospital: A Descriptive Cross-Sectional Study

**DOI:** 10.31729/jnma.7221

**Published:** 2022-02-28

**Authors:** Amrita Giri, Anshumala Joshi, Sama Shrestha, Ashlesha Chaudhary

**Affiliations:** 1 Department of Obstetrics and Gynecology, Nepal Medical College and Teaching Hospital, Jorpati, Kathmandu, Nepal; 2 Nepal Medical College and Teaching Hospital, Jorpati, Kathmandu, Nepal

**Keywords:** *body mass index*, *metabolic syndrome*, *polycystic ovary syndrome*

## Abstract

**Introduction:**

Metabolic syndrome in polycystic ovarian syndrome is associated with a long-term risk of developing type 2 diabetes mellitus and cardiovascular disease. This study aims to find the prevalence of metabolic syndrome among patients with polycystic ovarian syndrome presenting to a tertiary care hospital.

**Methods:**

A descriptive cross-sectional study was done on women attending the obstetrics and gynecology outpatient department of a tertiary care hospital from June 2020 to May 2021. A total of 106 women diagnosed with polycystic ovarian syndrome using Rotterdam criteria 2003 were recruited for the study and cases of metabolic syndrome was defined according to the modified American Heart Association/National Heart Lung and Blood Institute. Ethical approval was taken from the Institutional Review Committee of Nepal Medical College and Teaching Hospital (Reference number: 001-077/078). Convenience sampling was done. The collected data was entered and analyzed in Statistical Package for the Social Sciences version 21. Calculation of point estimate at 95% confidence interval was done along with frequency and proportion for binary data.

**Results:**

Among 106 women with polycystic ovarian syndrome, 50 (47.1%) had metabolic syndrome (37.59-56.60 at 95% Confidence Interval). The most common component of metabolic syndrome was low high-density lipoprotein cholesterol in 90 (84.9%) followed by central obesity in 60 (56.6%), hypertriglyceridemia in 47 (44.33%), high fasting sugar in 34 (32.07%), and high blood pressure in 14 (13.2%).

**Conclusions:**

The prevalence of metabolic syndrome among patients with the polycystic ovarian syndrome was similar to other studies done in similar settings.

## INTRODUCTION

Polycystic ovarian syndrome (PCOS) is a common gynecological endocrine disease among reproductive-aged women.^1^ According to Rotterdam criteria in 2003, PCOS is diagnosed when at least two of these three features i.e., oligo-ovulation, hyperandrogenism, and polycystic ovaries are present.^2^ The prevalence of PCOS is reported as 6.3% to 20.9%.^3^

Metabolic syndrome (MetS) is a web of risk factors associated with increased risk of diabetes mellitus and cardiovascular disease showing a 30-40% probability of developing diabetes and cardiovascular disease within the next 20 years.^4^ Peripheral insulin resistance and compensatory hyperinsulinemia contribute to androgen excess which is characteristic for both PCOS and MetS and obesity which is common to both amplify the risk.^5^ The prevalence of MetS in PCOS is reported as 43% in the United States, 33.8% in Germany, and 37.5% in India.^6-8^

The aim of this study was to find the prevalence of MetS in PCOS women presenting to a tertiary care hospital.

## METHODS

A descriptive cross-sectional study was carried out in the department of obstetrics and gynecology at Nepal Medical College and Teaching Hospital over the period of one year from June 2020 to May 2021 after taking ethical clearance from Institutional Review Committee (Reference number: 001-077/078). All the patients diagnosed with PCOS within the age group of 15-40 years were enrolled for the study after taking informed consent. Convenience sampling method was used. The sample size was calculated by using the formula,

n = Z^2^ × p × q / e^2^

  = (1.96)^2^ × 0.5 × (1-0.5) / (0.1)^2^

  = 97

Where,

n = minimum required sample size
Z = 1.96 at 95% Confidence Interval (CI)
p = Prevalence taken as 50% for maximum sample size.
q = 1-p
e = margin of error, 10%

Adding 10% for non-response rate to the calculated sample size, the calculated sample size was 106. Hence, a sample size of 106 women was taken for this study.

PCOS was diagnosed according to Rotterdam criteria 2003, when at least two of these three features were present namely oligo-ovulation or anovulation manifested as oligomenorrhea or amenorrhea, hyperandrogenism (clinical evidence of androgen excess) or hyper-androgenemia (biochemical evidence of androgen excess), and polycystic ovaries (defined on ultrasonography as the presence of 12 or more follicles in each ovary, measuring 2-9mm in diameter, and/or increased ovarian volume >10ml).^2^ Metabolic syndrome was diagnosed according to modified American Heart Association/National Heart Lung and Blood Institute AHA/NHLBI (ATP III 2005) as the co-occurrence of three or more of the following risk factors namely: i) waist circumference of ≥88cm or more, ii) blood pressure ≥130/85 mm Hg, iii) fasting blood sugar of ≥ 100mg/dl, iv) triglycerides of ≥ 150mg/ dl, and v) HDL of ≤50mg/dl.^9^

Women with established diabetes mellitus, thyroid dysfunction, hyperprolactinemia, and taking oral contraceptive pills and metformin were excluded from the study.

Each woman underwent a physical examination and laboratory evaluation for the diagnosis of metabolic syndrome. Weight was measured in kilograms to the nearest of 0.1kg and height was measured in cm to the nearest of 0.5cm. BMI was calculated as weight in kg divided by height in meter square. Blood pressure was measured in a sitting position in the right arm after 5 minutes of rest. Waist circumference was taken according to WHO in standing position, midway between the lowest rib and the iliac crest at the end of expiration, and hip circumference was taken around the widest portion of the buttock. The patient was then advised to do a blood sugar and lipid profile in fasting state along with other investigations (hormonal profile) required for PCOS and the results were entered in a Proforma.

If metabolic syndrome was diagnosed, or if an isolated rise of blood pressure, abnormality in lipid profile, or blood sugar was seen, then counseling regarding risks associated was done and the patient was advised for lifestyle modification e.g. diet modification and exercise. They were then referred to medicine OPD for further management.

Data was entered and analyzed in Statistical Package for the Social Sciences version 21. Point estimate at 95% Confidence Interval was calculated along with frequency and percentage for binary data.

## RESULTS

Among 106 women with polycystic ovarian syndrome, 50 (47.1%) had metabolic syndrome (37.59-56.60 at 95% Confidence Interval). The mean age of the patients of metabolic syndrome with PCOS was 26.72±5.31 years and the mean BMI was 27.08±5.37kg/m^2^. There were 30 (28.3%) women who met three criteria of metabolic syndrome, 16 (15.09%) met four criteria whereas four (3.77%) women had all five criteria of metabolic syndrome. There were only seven (6.6%) women who did not have any component of metabolic syndrome. The majority of women with PCOS had at least one abnormality of the MetS present 99 (93.39%) (Table 1).

**Table 1:** Prevalence of components of the metabolic syndrome in PCOS women (n = 106).

Metabolic syndrome components	n (%)
No component present	7 (6.60)
Only one component present	29 (27.35)
Two components present	20 (18.86)
Three components present	30 (28.3)
Four components present	16 (15.09)
All five components present	4 (3.77)

Among 106 women with PCOS, 42 (39.6%) were of normal weight whereas 32 (30.18%) were overweight and 30 (28.3%) were obese. Out of 62 overweight and obese women, with PCOS the prevalence of metabolic syndrome was 47 (75.8%) and out of 42 normal-weight women with PCOS, metabolic syndrome was seen in only three (7.14%).

The prevalence of metabolic syndrome increased with increasing age. Out of 51 young women with age less than 25, the rate of MetS was 19 (37.25%) which was alarmingly high (Table 2).

**Table 2:** Prevalence of metabolic syndrome in PCOS in different age groups.

Age	Total no. of patients (n= 106) n (%)	PCOS with MetS (n= 50) n (%)	PCOS PCOS without MetS (n= 56) n (%)
<19 years	10 (9.43)	3 (30)	7 (70)
20-24 years	41 (38.67)	16 (39.02)	25 (60.97)
25-29 years	38 (35.84)	18 (47.36)	20 (52.63)
30-34 years	13 (12.26)	9 (69.23)	4 (30.76)
>35 years	4 (3.77)	4 (100)	-
Mean±SD (years)	25.38±4.80	26.72±5.31	24.10±3.94

The BMI of the women in this study ranged from 18.2 to 47.3kg/m^2^. The prevalence of metabolic syndrome increased with increasing BMI (Table 3).

**Table 3:** Prevalence of Metabolic syndrome in PCOS according to BMI.

BMI (kg/m^2^)	PCOS with MetS (n= 50) n (%)	PCOS without MetS (n= 56) n (%)
<18.5	-	2 (100)
18.5-24.9	3 (7.14)	39 (92.85)
25-29.9	20 (62.5)	12 (37.5)
>30	27 (90)	3 (10)
Mean±SD of BMI	30.65±4.66	23.89±3.71

The most common component of metabolic syndrome seen in women with PCOS was HDL-C <50mg/dl 90 (84.9%) followed by central obesity which was seen in 60 (56.6%), hypertriglyceridemia 47 (44.33%), high fasting blood sugar 34 (32.07%) and high blood pressure 14 (13.2%) (Table 4).

**Table 4:** Prevalence of individual components of metabolic syndrome in PCOS.

Individual component	PCOS with MetS (n = 50) n (%)	PCOS without MetS (n= 56) n (%)
HDL <50mg/dl	48 (96)	42 (75)
Abdominal circumference >88cm	45 (90)	15 (26.78)
Triglycerides ≥150mg/dl	41 (82)	6 (10.71)
Fasting blood sugar ≥100mg	28 (56)	6 (10.71)
Blood pressure ≥130/85mm Hg	12 (24)	2 (3.5)

The age of the women, their BMI, waist/hip ratio, and all the components of metabolic syndrome in women with PCOS with metabolic syndrome are shown below (Table 5).

**Table 5:** PCOS women with metabolic syndrome.

Characteristics	With MetS (n= 50) (Mean±SD)	Without MetS (n= 56) (Mean±SD)
Age (year)	26.72±5.31	24.10±3.94
BMI (kg/m^2^)	30.65±4.66	23.89±3.71
Abd circumference (cm)	95.77±10.13	80.71±10.8
HDL level (mg/dl)	37.42±5.74	46.85±11.6
Triglyceride level (mg/dl)	194±12.19	103.40±4.34
Fasting blood sugar (mg/dl)	101.38±12.35	88.19±9.24
Systolic BP (mmHg)	121.6±12.67	111.6±8.48
Diastolic BP (mmHg)	76±10.10	71.07±4.54
Waist/hip ratio	0.89±0.06	0.85±0.06

## DISCUSSION

Metabolic syndrome is characterized by a cluster of factors including central obesity, hypertension, dyslipidemia and hyperglycemia all of which are the risk factors for cardiovascular disease mainly coronary heart disease and stroke. It also increases the risk of developing diabetes mellitus by fivefold. Though there is no established cure for PCOS, lifestyle changes have demonstrated substantial improvement in symptoms and complications.

The prevalence of metabolic syndrome among PCOS women in our study was 47.1% which was similar to 45.8% reported by Madhusudaran RR, et al.^10^ from India, 46% by Glueck, et al.^11^, 39.16% by Sharma S^12^ ,and as 43.4% by Ishak A, et al.^13^

The mean age of women with PCOS in our study was 25.38 years which is similar to that reported by Mandrelle K, et al.^8^ as 26.15 years and as 24.9 years by Carmina E, et al.^14^ The prevalence of metabolic syndrome increased with increasing age in our study and was seen in 37.25% of women less than 25 years which increased dramatically to 76.4% in women more than 30 years of age. In agreement with our study, Apridonidze T, et al.^15^ also found that with age the prevalence of metabolic syndrome increased from 23% in women less than 20 years to 45% in age 20 to 29 years and to 53% in women more than 30 years. Mandrelle K, et al.^8^ also stated the prevalence of metabolic syndrome increased with age and was seen in 48.9% of women aged 25 to 29 years. As the risk of metabolic syndrome in women with PCOS is high even at a young age, it is important to highlight the need for early and regular screening in PCOS women of all ages to decrease the cardio-metabolic risks.

In our study, high BMI was seen in patients with metabolic syndrome. The metabolic syndrome was seen in only 7.14% of women with normal BMI, whereas the same increased to 75.8% in overweight and obese PCOS women. Similar to our study, LP Cheung, et al.^16^ also reported that metabolic syndrome was more prevalent in overweight and obese women (41.3%) than in normal-weight women (0.9%). Ehrmann, et al.^5^ stated that obesity, a key determinant of insulin resistance appeared to have an independent effect on the risk of metabolic syndrome and women with high BMI had nearly 14-fold increased chance of having MetS than women with low BMI and in their study, none of the women with BMI less than 27kg/ m^2^ had metabolic syndrome. Similar to our study, Karee M, et al.^17^ found the most frequently observed individual components of MetS were increased waist circumference and decreased HDL cholesterol. As MetS is associated with increased BMI and BMI being modifiable, lifestyle modification with a reduction in weight can reduce the long-term risk of developing cardiovascular disease.

The most common component of MetS in our study was low HDL-C which was seen in 84.9% of patients followed by central obesity 56.6% and hypertriglyceridemia 44.33%. High triglycerides and low HDL-C are the characteristic types of dyslipidemia seen in insulin-resistant subjects. In agreement with our study, Apridonidze T, et al.^15^ reported that low HDL-C (68%) occurred most frequently in PCOS with MetS followed by elevated BMI (67%), high BP (45%), and hypertriglyceridemia (35%). The finding is also consistent with those of Legro RS, et al.^18^ who also reported a high prevalence of low HDL-C (91%) in PCOS women. Similarly, Dokra A, et al. also stated that among PCOS women, the most common feature of MetS was increased BMI, followed by low HDL-C and increased triglycerides.^19^ As HDL-C provides cardiovascular protection by direct endothelial effects, low HDL-C are associated with long term risk of increased cardiovascular disease mainly coronary artery disease. If MetS is identified early, the risks could be reduced mainly by lifestyle modification (diet modification, weight reduction, and exercise) and with the use of drugs.

The key limitation of this study could be the small sample size and sampling bias. Also, as this is a descriptive cross-sectional study, an association between the variables could not be made and causality could not be established. Further, the single-center nature of this study limits the generalizability of the findings warranting the need for higher studies to find out the real picture of prevalence, association, and causality among the Nepalese population.

## CONCLUSIONS

The prevalence of metabolic among patients with polycystic ovarian syndrome was similar to other studies done in similar settings. The incidence of PCOS is increasing in reproductive age women in recent years so indirectly the number of women with metabolic syndrome will also increase. As the metabolic syndrome confers an increased risk of cardiovascular disease and diabetes, PCOS should be considered a disease with significant public health implications and all women with PCOS should be screened for metabolic syndrome. Lifestyle modifications with proper dietary counseling and exercise and sometimes with the use of drugs, the prevalence of MetS can be reduced in these women thus decreasing the longterm cardiometabolic risks.

## References

[1] Kauffman RP, Baker TE, Baker VM, DiMarino P, Castracane VD (2008). Endocrine and metabolic differences among phenotypic expressions of polycystic ovary syndrome according to the 2003 Rotterdam consensus criteria.. Am J Obstet Gynecol..

[2] Rotterdam ESHRE/ASRM-Sponsored PCOS consensus workshop group. (2004). Revised 2003 consensus on diagnostic criteria and long-term health risks related to polycystic ovary syndrome (PCOS).. Hum Reprod..

[3] Yildiz BO, Bozdag G, Yapici Z, Esinler I, Yarali H (2012). Prevalence, phenotype and cardiometabolic risk of polycystic ovary syndrome under different diagnostic criteria.. Hum Reprod..

[4] Enas EA, Mohan V, Deepa M, Farooq S, Pazhoor S, Chennikkara H (2007). The metabolic syndrome and dyslipidemia among Asian Indians: a population with high rates of diabetes and premature coronary artery disease.. J Cardiometab Syndr..

[5] Ehrmann DA (2005). Polycystic ovary syndrome.. N Engl J Med..

[6] Essah PA, Nestler JE (2006). Metabolic syndrome in women with polycystic ovary syndrome.. Fertil Steril..

[7] Hahn S, Tan S, Sack S, Kimmig R, Quadbeck B, Mann K (2007). Prevalence of the metabolic syndrome in German women with polycystic ovary syndrome.. Exp Clin Endocrinol Diabetes..

[8] Mandrelle K, Kamath MS, Bondu DJ, Chandy A, Aleyamma T, George K (2012). Prevalence of metabolic syndrome in women with polycystic ovary syndrome attending an infertility clinic in a tertiary care hospital in south India.. J Hum Reprod Sci..

[9] Grundy SM, Cleeman JI, Daniels SR, Donato KA, Eckel RH, Franklin BA, American Heart Association; National Heart, Lung, and Blood Institute. (2005). Diagnosis and management of the metabolic syndrome: an American Heart Association/National Heart, Lung, and Blood Institute Scientific Statement.. Circulation..

[10] Madusudhanan RR, Nambisan B, Brahmanandan M, Radha S (2017). Study on the Prevalence and Characteristics of Metabolic Syndrome in Women of Reproductive Age Group with Polycystic Ovarian Syndrome.. J South Asian Feder Obst Gynae..

[11] Glueck CJ, Papanna R, Wang P, Goldenberg N, Sieve-Smith L (2003). Incidence and treatment of metabolic syndrome in newly referred women with confirmed polycystic ovarian syndrome.. Metabolism..

[12] Sharma S, Majumdar A (2015). Prevalence of metabolic syndrome in relation to body mass index and polycystic ovarian syndrome in Indian women.. J Hum Reprod Sci..

[13] Ishak A, Kadir AA, Hussain NHN, Ismail SB (2012). Prevalence and Characteristics of Metabolic Syndrome among Polycystic Ovarian Syndrome Patients in Malaysia.. Int J Collab Res Intern Med Public Health..

[14] Carmina E, Lobo RA (1999). Polycystic ovary syndrome (PCOS): arguably the most common endocrinopathy is associated with significant morbidity in women.. J Clin Endocrinol Metab..

[15] Apridonidze T, Essah PA, Iuorno MJ, Nestler JE (2005). Prevalence and characteristics of the metabolic syndrome in women with polycystic ovary syndrome.. J Clin Endocrinol Metab..

[16] Cheung LP, Ma RC, Lam PM, Lok IH, Haines CJ, So WY (2008). Cardiovascular risks and metabolic syndrome in Hong Kong Chinese women with polycystic ovary syndrome.. Hum Reprod..

[17] Karee M, Gundabattula SR, Sashi L, Boorugu H, Chowdhury A (2020). Prevalence of metabolic syndrome in women with polycystic ovary syndrome and the factors associated: A cross sectional study at a tertiary care center in Hyderabad, south-eastern India.. Diabetes Metab Syndr..

[18] Legro RS, Arslanian SA, Ehrmann DA, Hoeger KM, Murad MH, Pasquali R, Endocrine Society. (2013). Diagnosis and treatment of polycystic ovary syndrome: an Endocrine Society clinical practice guideline.. J Clin Endocrinol Metab..

[19] Dokras A, Bochner M, Hollinrake E, Markham S, Vanvoorhis B, Jagasia DH (2005). Screening women with polycystic ovary syndrome for metabolic syndrome.. Obstet Gynecol..

